# Beliefs About Pain in Pediatric Inflammatory and Noninflammatory Chronic Musculoskeletal Conditions: A Scoping Review

**DOI:** 10.1093/jpepsy/jsad046

**Published:** 2023-09-21

**Authors:** Danielle C Mountain, Syed Mustafa Ali, Daniela Ghio, Janet E McDonagh, Lis Cordingley, Rebecca R Lee

**Affiliations:** Centre for Epidemiology Versus Arthritis, Centre for Musculoskeletal Research, Division of Musculoskeletal and Dermatological Sciences, Faculty of Biology, Medicine and Health, Manchester Academic Health Science Centre, University of Manchester, UK; NIHR Manchester Biomedical Research Centre, Manchester University Hospital NHS Trust, Manchester Academic Health Science Centre, UK; Division of Psychology and Mental Health, Manchester Centre for Health Psychology, University of Manchester, UK; Centre for Epidemiology Versus Arthritis, Centre for Musculoskeletal Research, Division of Musculoskeletal and Dermatological Sciences, Faculty of Biology, Medicine and Health, Manchester Academic Health Science Centre, University of Manchester, UK; Division of Informatics, Imaging and Data Sciences, Centre for Health Informatics, Manchester Academic Health Sciences Centre, University of Manchester, UK; Division of Psychology and Mental Health, Manchester Centre for Health Psychology, University of Manchester, UK; Centre for Epidemiology Versus Arthritis, Centre for Musculoskeletal Research, Division of Musculoskeletal and Dermatological Sciences, Faculty of Biology, Medicine and Health, Manchester Academic Health Science Centre, University of Manchester, UK; NIHR Manchester Biomedical Research Centre, Manchester University Hospital NHS Trust, Manchester Academic Health Science Centre, UK; Centre for Epidemiology Versus Arthritis, Centre for Musculoskeletal Research, Division of Musculoskeletal and Dermatological Sciences, Faculty of Biology, Medicine and Health, Manchester Academic Health Science Centre, University of Manchester, UK; NIHR Manchester Biomedical Research Centre, Manchester University Hospital NHS Trust, Manchester Academic Health Science Centre, UK; Division of Psychology and Mental Health, Manchester Centre for Health Psychology, University of Manchester, UK; Centre for Epidemiology Versus Arthritis, Centre for Musculoskeletal Research, Division of Musculoskeletal and Dermatological Sciences, Faculty of Biology, Medicine and Health, Manchester Academic Health Science Centre, University of Manchester, UK; NIHR Manchester Biomedical Research Centre, Manchester University Hospital NHS Trust, Manchester Academic Health Science Centre, UK; Division of Psychology and Mental Health, Manchester Centre for Health Psychology, University of Manchester, UK

**Keywords:** adolescent, children, health beliefs, musculoskeletal, pediatric, pain, pain beliefs

## Abstract

**Objective:**

The Common Sense Self-Regulatory Model posits that beliefs about pain influence coping behaviors and subsequent physical and mental health outcomes in children/young people with chronic musculoskeletal conditions. It was unclear how and what beliefs had been investigated in this population, and whether there were similarities and differences in beliefs held about pain by those experiencing inflammatory versus noninflammatory musculoskeletal conditions. This scoping review addressed this gap.

**Methods:**

A systematic search was conducted using four databases (MEDLINE, PsycINFO, Embase, and CINAHL) in November 2021. Primary studies exploring key stakeholders’ (including children, parents, and/or healthcare professionals) beliefs about pain underlying pediatric chronic musculoskeletal conditions were synthesized.

**Results:**

Eighteen articles were identified. Cross-sectional designs were predominantly used to explore beliefs (*n* = 6). The majority used questionnaires to assess beliefs (*n* = 12). Beliefs common across musculoskeletal conditions were that children/young people felt their pain was not understood by others, and pain affected their physical functioning. Differences included children/young people and parents thinking they had some ability to control pain, and causal beliefs relating to underlying disease activity. These pain beliefs were more likely to be held in relation to inflammatory diagnoses. In contrast, children/young people and parents were more likely to view pain as uncontrollable, with more uncertainty regarding underlying causes, relating to noninflammatory diagnoses.

**Conclusions:**

Methods used to explore pain beliefs were inconsistent. Studies identified similarities and differences which appear to be closely related to the underlying diagnosis. Findings justify further exploration to identify potentially modifiable targets to improve pain outcomes in this population.

## Introduction

Between 4% and 40% of children/young people (defined as ages up to 24 years; McDonagh et al., 2018) have been identified as experiencing chronic musculoskeletal pain in the most recent epidemiological review synthesizing existing knowledge about the prevalence of chronic pain (King et al., 2011). Chronic pain is defined as pain that is persistent or recurrent for more than 3 months (Treede et al., 2019). The term chronic musculoskeletal pain specifically refers to pain within the joints, bones, muscles, and/or tendons (Perrot et al., 2019). Recently in the field of pain, distinctions between primary versus secondary pain were introduced (Treede et al., 2019). Despite this, healthcare professionals particularly in pediatric rheumatology, who manage a range of musculoskeletal conditions, appear to use alternative terminology in their clinical practice to differentiate between different types of pain, such as inflammatory and/or noninflammatory categories (Lee et al., 2020). Such distinctions appear to be related to different pain management approaches for those who have a perceived inflammatory diagnosis (e.g., medical management of disease activity) compared to those with a perceived noninflammatory diagnosis (e.g., referral to other services, including psychology or pain services). Irrespective of these categorizations, chronic pain can often continue to persist into adulthood if not addressed early on in childhood (Kashikar-Zuck et al., 2019) and significantly affects children/young peoples’ physical, psychological, and social functioning (Forgeron et al., 2010; Holley et al., 2017; O'Sullivan et al., 2011).

A recent study demonstrated that healthcare professionals within pediatric rheumatology (including rheumatologists, nurses, physical therapists, pediatricians, and occupational therapists) believe there are differences in the characterization of pain experiences between inflammatory versus noninflammatory musculoskeletal diagnoses (Lee et al., 2020). Pain experiences consist of biological, psychological, and social (biopsychosocial) factors (Gatchel et al., 2007), and beliefs predominantly belong to the cognitive psychological component of this wider experience. In the broader context of pain experiences, there is limited and inconsistent evidence on whether experiences are similar or different between pediatric inflammatory versus noninflammatory musculoskeletal conditions (Agoston et al., 2016; Eccleston et al., 2005; Fraga et al., 2019; Gauntlett-Gilbert et al., 2013; Vowles et al., 2010). In comparison to inflammatory conditions, noninflammatory conditions have been associated with poorer social, physical, emotional, and developmental functioning (Connelly et al., 2019; Eccleston et al., 2005), higher pain perception (pain intensity) (Connelly et al., 2019; Fraga et al., 2019), and individuals are less likely to use key pain-coping methods (e.g., problem-solving or seeking social support) (Fraga et al., 2019).

Beliefs are defined as “something that is accepted, considered to be true, or held as an opinion” (Merriam-Webster, n.d.) and are determined from current and previous bodily experiences as well as the social context (e.g., beliefs of authoritative figures such as parents and/or healthcare professionals) (Asmundson et al., 2012; Leventhal et al., 1980). In relation to pain, beliefs are important as they influence how a person manages, behaves, or responds to pain. For example, beliefs that are considered to be “unhelpful” can lead to avoidance of activities, such as believing pain always corresponds to damage in the body (Caneiro et al., 2021). A prominent cognitive model that captures and theorizes these links is the Common Sense Self-Regulatory Model (CS-SRM) (Leventhal et al., 1980, 2012). The CS-SRM provides a framework for understanding how “representations” of health directly or indirectly influence pain perception, pain-coping methods, and health outcomes (Leventhal et al., 1980, 2012). According to the CS-SRM, illness representations that depict high levels of threat, such as serious consequences or a chronic timeline, can lead to maladaptive pain-coping methods (Hagger et al., 2017).

Beliefs are modifiable and therefore can be useful targets for therapeutic intervention, which aim to improve children/young peoples’ health outcomes. For example, cognitive behavioral therapy (CBT) is a psychological therapy that can specifically be applied to and used in the context of pain to explain and re-frame pain beliefs and behaviors (Caes et al., 2018). CBT has been associated with improved health-related quality of life and reduced pain, pain catastrophizing, and disability in children/young people with chronic pain (Fisher et al., 2018; Lomholt et al., 2015; Palermo et al., 2010). However, further research is needed to explore children/young peoples’ pain beliefs in order to inform treatment targets and components of future interventions in this area. The CS-SRM is a valuable cognitive model for this, as it has been effective in structuring the evaluation of health beliefs in other pediatric long-term health conditions, such as asthma and attention deficit hyperactivity disorder (Tiggelman et al., 2014; Wong et al., 2018), with emerging evidence supporting the utility of the CS-SRM in the context of pediatric pain (Ghio et al., 2018). Therefore, the CS-SRM provided the theoretical framework for this scoping review. It addresses a broad range of interconnected pain beliefs that may be held by an individual, knowledge of which is fundamental for informing treatment targets that can be modified through interventions (such as CBT) to improve pain-coping behaviors and health outcomes (Leventhal et al., 2012).

To our knowledge, no studies to date have synthesized existing research findings and identified gaps in literature exploring pain beliefs associated with different children/young peoples’ musculoskeletal conditions, such as inflammatory versus noninflammatory conditions. This is important to address as healthcare professionals believe these are the most clinically relevant phenotypes, phenotypes which, in turn, appear to drive differences in the management of pediatric musculoskeletal pain. The aim of this scoping review was to synthesize research evidence on how pain beliefs have been examined in relation to children/young people with musculoskeletal conditions, particularly identifying theoretical and methodological components to existing studies. The aim of this scoping review was also to determine similarities and differences in pain beliefs between pediatric musculoskeletal condition types (inflammatory and/or noninflammatory). The results of this will inform interventions (e.g., CBT) to modify unhelpful beliefs that are shared or different between pediatric chronic musculoskeletal conditions, and subsequently improve children/young peoples’ health and pain-related outcomes.

## Materials and Methods

### Scoping Review

A scoping review exploring pain beliefs associated with children/young peoples’ musculoskeletal conditions was undertaken. A scoping review was deemed most suitable for identifying, synthesizing, and mapping the current extent of research and study findings on this specific topic area and for highlighting gaps in literature (Arksey & O'Malley, 2005). The five scoping review stages were followed, which are recommended by Arksey and O'Malley (2005) and Joanna Briggs Institute guidelines (Peters et al., 2020). The Preferred Reporting Items for Systematic Reviews and Meta-Analyses Extension for Scoping Reviews (PRISMA-ScR) (Tricco et al., 2018) was applied in the reporting of this review (Supplementary Table 1). The scoping review protocol was published on Open Science Framework (https://osf.io/hpt5a/).

### Search Strategy

A systematic search of four databases was conducted on November 30, 2021 (MEDLINE, Embase, and PsycInfo via Ovid, and CINAHL via Ebsco). Databases were searched from ever—2021 and restricted to English language manuscripts. The search strategy was developed and refined through multiple meetings between the author team (D. C. Mountain, J. E. McDonagh, L. Cordingley, and R. R. Lee) and a specialist academic librarian. Key words and Medical Subject Headings (MeSH) were chosen and applied to capture the relevant age (e.g., children and young people), diagnosis (e.g., musculoskeletal), pain type (e.g., persistent or episodic pain), and pain beliefs. Terms were combined using Boolean operators (“AND”, “OR”) and the search strategy was appropriately amended for each database. Handsearching of reference lists of relevant articles was conducted. References were retrieved from the databases and imported to EndNote (X9.2, 2019) (Clarivate Analytics, PA, USA) for removal of duplicates. The components of the full search strategy mapped onto both research aims and is provided in Supplementary Table 2.

### Study Selection

Studies were deemed eligible for inclusion if they (a) explored beliefs about pain associated with a diagnosed pediatric musculoskeletal condition (including beliefs of children/young people and those involved in their medical management, that is, parents/caregivers and healthcare professionals), (b) the following diagnoses: JIA, systemic lupus erythematosus (SLE), joint hypermobility syndrome, idiopathic pain syndromes, and back pain, and (c) were peer-reviewed primary research. Studies that grouped participants into general “chronic pain” samples (e.g., JIA participants combined with abdominal pain and cancer pain participants) were not included if data relating to the chronic musculoskeletal condition could not be readily extracted. Alternative terminology for eligible diagnostic labels was included (e.g., complex widespread pain). Back pain was only eligible if it could be differentiated as inflammatory (i.e., related to inflammatory arthritis) or noninflammatory (i.e., nonspecific chronic low back pain) due to difficulties and complexities of determining the underlying pathology of back pain (Cuthbert, 2019; Frosch et al., 2022). This distinction was provided either by the papers or through discussions with an experienced consultant pediatric rheumatologist (20+ years) within the scoping review team (J. E. McDonagh). The criteria for the musculoskeletal conditions were refined to those most routinely seen in pediatric and adolescent rheumatology (Clinch & Eccleston, 2009; Davies & Copeman, 2006; Rosenberg, 2005), and confirmed and discussed with a pediatric rheumatologist within the author team (J. E. McDonagh). The full inclusion and exclusion criteria are displayed in Table 1.

**Table 1.** Eligibility Criteria

Inclusion Criteria	Exclusion Criteria
Pain beliefs associated with chronic pediatric musculoskeletal diagnoses. Beliefs of the children/young people and those involved in their medical management (i.e., parents/caregivers and healthcare professionals). Diagnoses in ages up to 24 years or below. For samples that have participants up to 24 years of age, the majority of the sample must be aged 19 years or below (i.e., mean and standard deviation) to capture adolescence (i.e., ages 10–19 years) (WHO, 2002). Beliefs in relation to inflammatory musculoskeletal diagnoses (JIA and SLE, back pain) and noninflammatory diagnoses (joint hypermobility syndrome, including Hypermobile Ehlers Danlos type III, idiopathic pain, and back pain). Peer-reviewed primary research. Quantitative, qualitative or mixed-methods. Experimental or observational research. English language.	Beliefs about the illness as a whole (e.g., beliefs about JIA). Children/young people diagnosed for the purpose of the study or who did not have a clinical diagnosis. Studies where the data of the specified ages or relevant musculoskeletal conditions were unable to be easily and readily extracted. Experimental studies whereby the pre-intervention (i.e., baseline) data was not easily or readily extractable. Development or validation of psychometric tools. Nonprimary research (e.g., dissertations), case reports, conference abstracts. Pain catastrophizing that is referred to as a coping mechanism (e.g., the catastrophizing subscale of the Coping Strategies Questionnaire)

*Note.* JIA = juvenile idiopathic arthritis.

Manuscripts were exported from EndNote (2019) into a Microsoft Excel Spreadsheet. Two reviewers (D. C. Mountain and S. M. Ali) pilot screened 100 titles and abstracts to assess consensus and understanding of the eligibility criteria and they then screened all titles and abstracts. The full texts of articles that met the inclusion criteria or that were unclear were obtained and screened against the eligibility criteria. The two reviewers were blinded to each other’s decisions throughout screening. Disagreements were discussed between the two reviewers and if unresolved then these were discussed with a third reviewer (L. Cordingley or D. Ghio).

Existing literature assessing pain beliefs uses heterogeneous approaches to explore beliefs, such as studies using no theoretical models or a range of models. The CS-SRM (Leventhal et al., 1980, 2012) is a theoretical framework that was used during selection. Comparisons of cognitive models have identified the CS-SRM’s illness representations as the most informative predictor of the impact of cognitive and emotional beliefs, such as identity, timeline, and control, on an individual’s functioning in conditions such as low back pain (Bishop et al., 2015; Foster et al., 2010). The CS-SRM comprises beliefs about pain *identity* (e.g., the label and symptoms associated with pain), *causal beliefs*, *consequences* of pain, *controllability* of pain, *timeline* (e.g., how the pain changes across time and chronicity), *coherence* (e.g., understanding of pain), and *emotional representation* beliefs (e.g., distress associated with pain). The Illness Perception Questionnaire-Revised (IPQ-R) (Moss-Morris et al., 2002) is a psychometric tool that measures the CS-SRM’s illness representations. The IPQ-R provides a list of belief statements for each illness representation that acted as a frame of reference during study selection to identify where study findings fit with the CS-SRM (e.g., *consequences*: “my pain has major consequences on my life” or “my pain does not have much effect on my life”). Pain catastrophizing and kinesiophobia (i.e., a fear of movement or reinjury due to pain) have overlapping features with the CS-SRM, such as emotional representations and beliefs of consequences. Therefore, these were included in the study selection. The use of the term “pain catastrophizing” is inconsistent in the literature, either being used as a belief or a behavior therefore studies that used coping-related pain catastrophizing were not eligible for inclusion in the current scoping review.

### Charting the Data

A standardized Microsoft Excel Spreadsheet was used to extract data from the manuscripts included in this review. Two reviewers (D. C. Mountain and S. M. Ali) independently extracted data from 25% of the eligible articles to ensure the data extracted were relevant and consistent across both reviewers. The two reviewers discussed any disagreements together or with another review member (D. Ghio or L. Cordingley). Following this, one reviewer (D. C. Mountain) extracted data from all remaining articles. Data extracted was based upon the research team’s previous experience of data extraction for reviews and adapted to reflect the current aims. Data included authors, year of publication, country, aims, study sample (e.g., sample size, age, gender), method (quantitative, qualitative, or mixed-methods), design (e.g., cross-sectional, longitudinal), pain belief data collection measure used (e.g., questionnaire, interview), pain belief source (e.g., children/young people self-report, parent self-report or healthcare professional self-report), sample’s musculoskeletal diagnosis pathology (inflammatory, noninflammatory, mixed), diagnosis (e.g., JIA), explicit use of a theoretical framework in their introduction or methods (including the name of the theory), and key findings (e.g., descriptions of beliefs relating to the CS-SRM’s illness representations). During extraction, the authors discussed and confirmed the relevance of research findings by comparing these to the pain belief statements from the IPQ-R. This allowed a method of defining illness representations and mapping the findings to the CS-SRM. Expert consensus was reached within the team of experienced health psychologists and a pediatric rheumatologist by discussing the extracted findings during multiple team meetings to ensure the appropriate information was extracted. Social beliefs (e.g., children/young peoples’ belief that family should behave a certain way toward their pain; solicitude subscale), pain education beliefs (e.g., understanding of what pain signals and whether exercise is beneficial; exercise and harm subscales) and concerns about adherence to treatment (e.g., beliefs about the prescription of and adherence to pain medication; medication subscale) were deemed beyond scope of the review and were not extracted.

Where possible, the reviewers extracted the author’s description of their study design. However, in some instances, the reviewer had to determine the most suitable description for the study design (e.g., if a qualitative study did not explicitly state the research design, then the researcher determined this based upon their methods, such as whether it took a phenomenological design or ethnographic design). Only baseline data were extracted for experimental studies. A mixed-diagnosis sample was defined as either containing participants who had a mixed-diagnosis (e.g., a child diagnosed with both JIA and idiopathic pain) or containing a sample of both inflammatory or noninflammatory diagnoses. If the data of inflammatory and noninflammatory diagnoses could be readily extracted from a mixed sample, then this was done. However, in samples where this was not possible, the results were described as “mixed.”

### Collating, Summarizing, and Charting the Results

Descriptive statistics and a summary chart were used to detail how the studies were conducted. A narrative synthesis and summary chart highlight (a) how pain beliefs have been explored, and (b) what pain beliefs were associated with inflammatory, noninflammatory, and mixed-diagnosis samples. The CS-SRM states that illness representations are not independent domains but instead interact and interlink (Leventhal et al., 2012). As the eligible manuscripts were not explicitly underpinned by the CS-SRM their findings were less specific to one domain of illness representations (i.e., a finding could map across interlinking illness representations). It is for these reasons that the authors deemed it appropriate to identify their own conceptual headings for the narrative synthesis of what beliefs were associated with each diagnosis. “Beliefs about and understanding of pain cause” reflected CS-SRM beliefs of coherence, cause, and identity, “beliefs about the impact of pain” reflected CS-SRM beliefs of consequences and emotional representations, and “beliefs about pain controllability and pain management” represented CS-SRM beliefs of control and timeline.

Quantitative results are described in the text if the papers provided an interpretation of the score (i.e., what cut-off score is defined as low/moderate/high and the context of this, that is, whether a high score is positive or negative) or, to the author’s knowledge, clinical reference points have been determined in the literature. The following cut-offs were used for the Pain Catastrophizing Scale (PCS), the PCS for Children (PCS-C), and the Pain Self-Efficacy Questionnaire (PSEQ). PCS scores: low = 0–19, moderate = 20–29, high = 30 and above (Sullivan, 1995). PCS-C scores: low = 0–14, moderate = 15–25, high = 26 and above (Pielech et al., 2014). PSEQ scores: low ≤ 30, high ≤ 40 (Nicholas, 2007). An illustrative diagram (Figure 2) displays an example of children/young peoples’ illness representations that have been assessed in relation to inflammatory and noninflammatory musculoskeletal conditions.

## Results

### Study Selection

**Figure 1.**
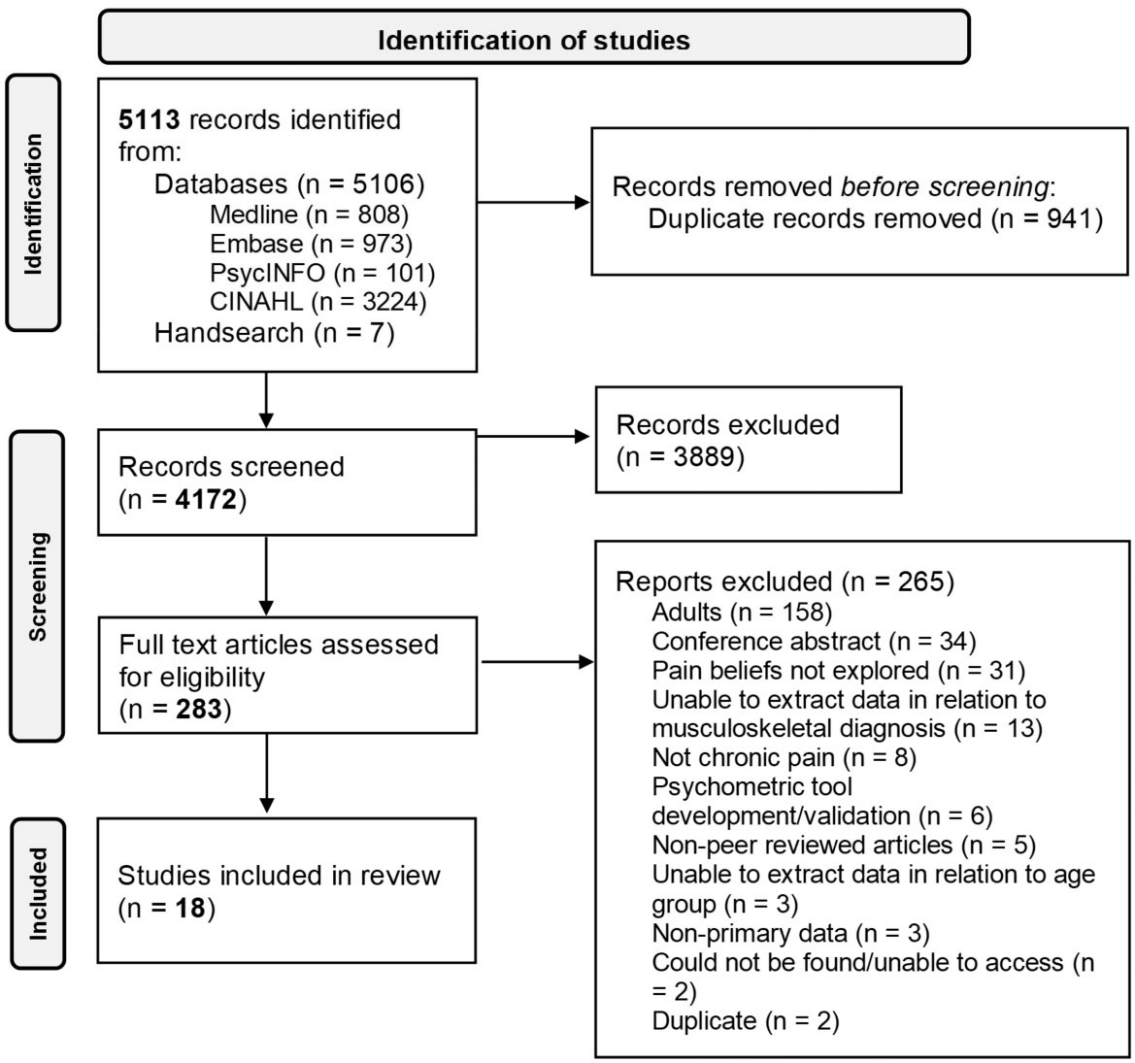
PRISMA flow diagram.

A total of 5113 records were identified from database searches and hand searches. Nine hundred forty-one duplicates were removed. Four thousand one hundred seventy-two sources underwent title and abstract screening. Two hundred eighty-three full texts were screened. Eighteen articles were identified as meeting the criteria for the scoping review (Figure 1).

### Study Methods and Design

Included articles were published between the years 2000 and 2020 and conducted in eight countries, with the majority taking place in the United States (*n* = 7) [10, 11, 13–17]. The majority of studies were cross-sectional in design (*n* = 6) [1, 5, 13–16] and predominantly assessed pain beliefs in either an inflammatory musculoskeletal diagnosis sample (*n* = 8) [1–8] or noninflammatory diagnosis sample (*n* = 8) [9–16]. Sample sizes ranged from 6 to 274 participants. The method of reporting participants’ ages varied between studies, however of that which were described, the reported mean ages of children/young people were 11.4–20.8 years and reported age ranges were 7–24 years. The percentage of children/young people who were female was between 50% and 90% and parent self-reporters tended to predominantly be mothers (62%–92%). Six manuscripts [10, 11, 13–16] reported participants’ race and/or ethnicity and most participants were White or Caucasian (68%–92.5%). The majority of studies recruited participants from clinical settings, such as pediatric rheumatology (*n* = 7) [2–4, 6–8, 10] and pain clinics (*n* = 6) [10, 12–16]. A summary of study characteristics is located in Table 2.

**Table II.** Summary of Included Studies’ Characteristics

Study Characteristics	Study Code		
	Inflammatory	Noninflammatory	Mixed
Year			
2000–2004	1		
2005–2009	7	11, 13	
2010–2014	3, 8	9, 14, 15, 16	17
2015–2019	4, 5, 6	10, 12	18
2020	2		
Country			
United States		10, 11, 13, 14, 15, 16	17
Canada	6		
England	2		
Republic of Ireland	2		
Australia		9	18
Norway		12	
Denmark	3, 4, 5, 7, 8		
Finland	1		
Design			
Phenomenological	2, 6	12	17
Grounded theory		11^b^	
Cross-sectional	1, 5	13, 14, 15, 16	
Case-control		9	
Cohort/longitudinal	3^a^, 7^a^, 8^b^		
Randomized control trial		10	
Nonrandomized control trial	4		
Mixed-methods			18
Diagnosis			
JIA/JRA	1, 2, 3, 4, 5, 6, 7, 8		
NSCLBP		9	
Juvenile fibromyalgia		10, 11	
CRPS		11, 12,	
≥3 noninflammatory diagnoses		13, 14, 15, 16	
Mixed-diagnosis sample			17, 18

*Note*. Study codes. 1–8 inflammatory sample, 9–16 noninflammatory sample, 17–18 mixed-diagnosis: 1 Kyngäs (2000); 2 Lee et al. (2020), 3 Lomholt et al. (2013); 4 Lomholt et al. (2015); 5 Nørgaard et al. (2017); 6 Race et al. (2016); 7 Thastum et al. (2005); 8 Thastum and Herlin (2011); 9 Astfalck et al. (2010); 10 Kashikar-Zuck et al. (2018); 11 Meldrum et al. (2009); 12 Sørensen and Christiansen (2017); 13 Guite et al. (2009); 14 Guite et al. (2011); 15 Guite et al. (2014b); 16 Guite et al. (2014a); 17 Gaughan et al. (2014); 18 Slater et al. (2016).

CRPS = Complex Regional Pain Syndrome; JIA = juvenile idiopathic arthritis; JRA = juvenile rheumatoid arthritis; NSCLBP = nonspecific chronic low back pain.

a Pain beliefs only explored cross-sectionally.

b Pain beliefs explored at two time points.

### Pain Belief Data Collection Approaches

Table 3 presents descriptions of how pain beliefs were assessed.

**Table III.** Pain Belief Data Collection Methods Used in Relation to Pediatric Chronic Musculoskeletal Conditions

Study Code	Author (s)	Year	Sample Size	Study Sample Characteristics (Age, Gender, Race, Ethnicity)	Recruitment Setting	Pain Belief Elicitation Method	Pain Belief Respondent	Purpose of the Pain Belief Assessment
Inflammatory Musculoskeletal Samples (1–8)								
1	Kyngäs	2000	274	*M* age = 14.6 ± 1.39 years 146 female Race/ethnicity NR	Social Insurance Institution Register	Questionnaire made for the purpose of the study	CYP self-report	Explored the relationship between the belief of “control” and treatment adherence
2	Lee, Rashid, Thomson and Cordingley	2020	21	Age NR 19 female Race/ethnicity NR	Pediatric rheumatology	Semi-structured interview	Healthcare professional self-report	Explored pediatric rheumatology healthcare professionals’ beliefs about the role of pain and pain assessment in JIA
3	Lomholt, Thastum, and Herlin	2013	91	*M* age = 13.8 ± 2.4 years 21 male Race/ethnicity NR	Pediatric rheumatology	SOPA—Children’s version (Jensen et al., 1994) 4-point Likert scale	CYP self-report	Compared beliefs of “control” and “disability” between participants with pain or with no pain during a 2 week pain diary
4^a^	Lomholt et al.	2015	19	*M* age = 11.7 ± 1.7 years 15 female Race/ethnicity NR	Pediatric rheumatology	SOPA—Children’s version (Thastum et al., 2005) 3-point Likert scale (scale of 1–3)	CYP self-report	Explored whether beliefs of “control” and “disability” changed following treatment (cognitive behavioral therapy) compared to a waitlist group
5	Nørgaard et al.	2017	61	*Mdn* age = 13.3 years (12–14.4) 37 female Race/ethnicity NR	Outpatient clinic	SOPA—Children’s version (Engel et al., 2012; Thastum et al., 2005) Scale not described	CYP self-report	Explored the relationship between beliefs about “control,” “disability,” and “emotion” (e.g., emotional causal factors) and physical activity
6	Race et al.	2016	23 CYP 29 parents	*M* age = 11.9 ± 2.6 years 15 female CYP, 21 mothers Race/ethnicity NR	Pediatric rheumatology	Semi-structured interview	CYP and parent self-report	Explored perspectives of having JIA and the perceived barriers and facilitators to physical activity
7	Thastum, Herlin, and Zachariae	2005	56	*M* age = 11.4 ± 2.1 years 45 female Race/ethnicity NR	Pediatric rheumatology	SOPA—Children’s version (Jensen et al., 1994) Scale of 0-4, 0 = *very untrue*, 4 = *very true*	CYP self-report	Explored relationships between pain and beliefs of “control,” “disability,” and “emotion” (i.e., causal). Compared beliefs between children/young people with high pain but low disease activity with the rest of the sample
8	Thastum and Herlin	2011	47	Low disease activity group (age = 10.6 ± 2.1 years, 9 female) Remaining participants (age = 11.4 ± 2.1 years, 13 female) Race/ethnicity NR	Pediatric rheumatology	SOPA—Children’s version (Thastum et al., 2005) Scale of 0–4	CYP self-report	Assessed beliefs of “control,”, “disability,” and “medical cure” (i.e., treatment control) at baseline and 2 years later. Compared beliefs between children/young people with high pain but low disease activity with the rest of the sample)
Noninflammatory Musculoskeletal Samples (9–16)								
9^b^	Astfalck et al.	2010	28	*M* age = 15.4 ± 0.5 years 14 female Race/ethnicity NR	Longitudinal health research project—the Western Australian Pregnancy Cohort Study	Tampa Scale of Kinesiophobia (Vlaeyen et al., 1995) Scale not described	CYP self-report	Conducted a biopsychosocial characterization of nonspecific chronic low back pain compared to controls (pain-free)
10^a^	Kashikar-Zuck et al.	2018	40	*M* age = 15.38 ± 1.53 years 36 female Race (Caucasian *n* = 37, American Indian/Alaskan Native *n* = 2, >1 race *n* = 1) Ethnicity (non-Hispanic *n* = 40)	Pediatric rheumatology and pain clinics	Tampa Scale of Kinesiophobia - 11 (Tkachuk & Harris, 2012) Scale of 1–4, 1 = *strongly disagree*, 4 = *strongly agree* Pain Catastrophizing Scale for Children (Crombez et al., 2003) Scale of 0–4, 0 = *not at all*, 4 = *extremely*	CYP self-report	Assessed whether beliefs of fear of movement and pain catastrophizing changed at 3 months following either cognitive behavioral therapy or the fibromyalgia integrative training program for teens (FIT Teens) intervention
11	Meldrum, Tsao, and Zeltzer	2009	53	*M* age = 14.23 years 36 female Race (68% Caucasian, 15% Latino, 9% mixed race, 8% African American, Asian American, or other)	Tertiary university-based pediatric clinics	Semi-structured life history interview	CYP self-report	Explored children’s/young peoples’ experiences of pain and its impact on their live, including dysfunction, treatment and outcomes
12	Sørensen and Christiansen	2017	6	Age range = 12–19 4 female Race/ethnicity NR	Pain clinic	Semi-structured interview	CYP self-report	Explored children’s/young peoples’ experiences of complex persistent pain, including the impact this has on life
13	Guite et al.	2009	138	*M* age = 15.6 ± 1.3 years 116 female CYP, 127 mothers CYP ethnicity (white n = 120) Parent ethnicity NR	Pediatric pain clinic	Questionnaire made for the purpose of the study	Parent self-report	Explored relationships between parents’ beliefs of their child’s pain with their child’s pain and disability, parental pain promoting behaviors and distress, and family functioning. Beliefs included (a) pain was “medical only,” (b) parents felt “responsible” for children/young peoples’ pain, and (c) how much parents believed their child’s physical health, including pain, and emotional well-being caused parental worry/concern
14	Guite et al.	2011	138	*M* age = 15.6 ± 1.3 years 116 female CYP, 127 mothers CYP ethnicity (White *n* = 120) Parent ethnicity NR	Pediatric pain clinic	Pain Catastrophizing Scale (Sullivan et al., 1995) 5-point Likert scale (0–4)	CYP self-report	Explored relationships between pain catastrophizing with pain intensity, functional disability and children/young peoples’ perception of protective parental response
15	Guite et al.	2014b	102 CYP-parent dyads	*M* age = 15.7 ± 1.4 years 81 female CYP, 91 mothers CYP ethnicity (Caucasian *n* = 90) Parent ethnicity NR	Tertiary care center specialized for pediatric chronic pain	Questionnaire made for the purpose of the study	CYP and parent self-report	Explored relationships between children/young peoples’ and parents’ pain beliefs (causal factors of damage or emotion) with children/young peoples’ and parents’ pain treatment expectations (fictional vignette of a young person with chronic pain)
16	Guite et al.	2014a	102 CYP-parent dyads	*M* age = 15.7 ± 1.4 years 81 female CYP, 91 mothers CYP ethnicity (Caucasian *n* = 90) Parent ethnicity NR	Tertiary care center specialized for pediatric chronic pain	Questionnaire made for the purpose of the study Pain Catastrophizing Scale for Children (Crombez et al., 2003) 5-point Likert scale, 0 = *not at all*, 4 = *extremely* Pain Catastrophizing Scale for Parents (Goubert et al., 2006) 5-point Likert scale, 0 = *not at all*, 4 = *extremely*	CYP and parent self-report	Explored relationships between children/young peoples’ and parents’ pain beliefs (pain catastrophizing and causal factors of damage or emotion) with readiness to change self-management
Mixed-Diagnosis Samples (17–18)								
17	Gaughan et al.	2014	13	Age range = 11–17 years 8 female CYP, 8 mothers Race/ethnicity NR	Pediatric pain rehabilitation program	Semi-structured interview	Parent self-report	To explore parents’ beliefs of their child’s pain journey, including from initial onset to response to treatment and outcomes
18^b^	Slater et al.	2016	13	Age range = 18-24 years 10 female Race/ethnicity NR	Community settings (e.g., arthritis consumer organizations, mental health services)	Interview or focus group Pain Self-Efficacy questionnaire (Nicholas, 2007) 7-point Likert scale, 0 = *not at all confident*, 6 = *completely confident*	CYP self-report	To explore children/young peoples’ beliefs about persistent musculoskeletal pain, including what children/young people perceive they need to manage their condition

*Note.* CYP = children and young people; *M* = mean; *Mdn* = median; NR = not reported; SOPA = Survey of Pain Attitudes.

a Only baseline data has been extracted as this was an experimental study.

b Data reported from participants with eligible diagnoses (i.e., pain sample or have a diagnosis for their musculoskeletal condition). Questionnaire scales of validated questionnaires are as described by the author.

#### Pain Belief Reporter

The majority of manuscripts assessed solely children/young peoples’ self-reported pain beliefs (*n* = 12) [1, 3–5, 7–12, 14, 18]. Three studies assessed self-reported beliefs of both the parent and children/young people [6, 15, 16], two studies assessed parent’s self-reported beliefs [13, 17], and one explored pediatric rheumatology healthcare professionals’ pain beliefs [2].

#### Use of Theoretical Frameworks

Six manuscripts explicitly reported using theoretical frameworks [1, 12, 13–16]. The most commonly used were the “Biopsychosocial Model” (*n* = 3) [12, 15, 16] (Engel, 1980, 2012; Gatchel et al., 2007) and “Palermo and Chamber’s Integrative Model of Parent and Family Factors”(*n* = 3) [13, 15, 16] (Palermo & Chambers, 2005).

#### Pain Belief Data Collection Tools

The majority of studies used questionnaires (*n* = 12) [1, 3–5, 7–10, 13–16] to explore pain beliefs. Commonly used questionnaires included the *Survey of Pain Attitudes—Children’s version* (*SOPA-C*) (*n* = 5) [3, 4, 5, 7, 8] (Engel et al., 2012; Jensen et al., 1994; Thastum et al., 2005), the *Tampa Scale for Kinesiophobia* (*n* = 2) [9, 10] (Tkachuk & Harris, 2012; Vlaeyen et al., 1995), the *Pain Catastrophising Scale for Children* (*PCS-children*) (*n* = 2) [10, 16] (Crombez et al., 2003), and questionnaires specifically created for the purpose of the particular studies included (*n* = 4) [1, 13, 15, 16]. Questionnaires that were specifically created for studies included an assessment of children/young peoples’ compliance with managing their chronic musculoskeletal condition [1]; a brief assessment of parental beliefs and worries that related to children/young peoples’ chronic pain (e.g., that pain is medical only or that the child’s pain caused the parent concern) [13]; and a brief assessment of the degree to which children/young people and parents believed that pain is a biopsychosocial concept [15, 16].

### Pain Beliefs

**Figure 2.**
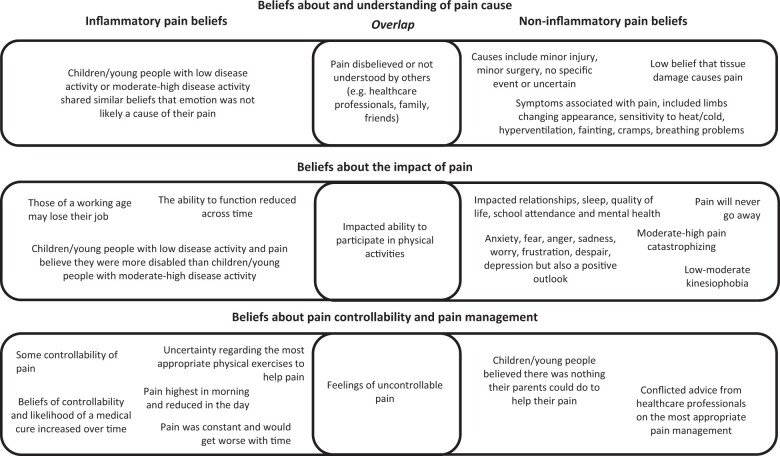
Similarities and differences in children’s/young peoples’ illness representations and outcomes between musculoskeletal conditions.

In the following sections, children/young peoples’ beliefs about pain that were found to be associated with inflammatory and noninflammatory chronic musculoskeletal conditions are discussed. Studies with mixed-diagnosis samples (*n* = 2) are combined and described within these sections. Similarities and differences in beliefs between children/young people and their parents and/or healthcare professionals are presented. Figure 2 provides an illustration of similarities and differences in children/young peoples’ beliefs about pain which appeared to be different depending on the underlying musculoskeletal diagnosis.

#### Inflammatory Musculoskeletal Conditions

Results below describe children/young peoples’ beliefs about the pain that underlie inflammatory musculoskeletal conditions (*n* = 8), which were predominantly focused on JIA or juvenile rheumatoid arthritis (JRA). A narrative account of two manuscripts [4, 5] could not be conducted due to no contextual descriptive information being available, such as questionnaire scores without a clinical reference point (e.g., low/moderate/high belief). The details of these studies are however included in Table 3, which presents descriptions of how pain beliefs were assessed.

##### Beliefs About and Understanding of Pain Cause

Children/young people with JIA and either low disease activity or moderate-high disease activity both shared similar beliefs that emotion was not likely a cause of their pain [7]. Regarding potential causes of pain, some pediatric rheumatology healthcare professionals perceived there being differences in causes of pain in JIA [2]. Biomedical physical causes were typically associated with pain, such as disease activity, whereas the credibility of the cause of persistent pain was questioned or perceived to be related to other factors, such as over-focusing on pain [2]. Children/young people reported feeling that healthcare professionals were disinterested in their pain or brushed their pain off [18]. Healthcare professionals also believed that some other healthcare professionals did not believe or understand children/young peoples’ pain [2]. Similarly, parents of children/young people with mixed-diagnoses believed that other people did not understand or believe their child’s pain, including friends and family [17]. Healthcare professionals were uncertain about the seriousness or relevance of children/young peoples’ chronic pain, such as believing pain should be proportionate to disease activity or believing that persistent pain in children/young people with JIA is unlikely to be caused by their arthritis [2]. Instead, healthcare professionals labeled pain that continued after disease activity was controlled (i.e., using pharmacological methods) as “chronic pains” [2].

##### Beliefs About the Impact of Pain

Children/young people believed their pain affected their ability to participate in physical activities [6, 18] and, for those of a working age, that pain may result in them losing their jobs [18]. Children/young people from a mixed-diagnosis sample reported that pain impacted their mental health and their ability to keep up with friends [18]. Not all young people with a mixed-diagnosis believed their pain affected their ability to work [18]. Interestingly, there was evidence that children/young peoples’ beliefs of the impact of pain increased over time (two years), including believing they were less able to function [8]. These beliefs appeared to be higher (i.e., stronger belief of disability) for children/young people with JIA and low disease activity compared to children/young people with JIA and moderate-high disease activity [7, 8]. Similarly, children/young people who reported pain during a two-week pain diary had a higher belief that they felt disabled due to their pain compared to children/young people who reported no pain [3]. Children/young peoples’ pain impacted healthcare professionals, as healthcare professionals reported how they (i.e., the healthcare professional) felt anxious by pain underlying JIA, particularly regarding the professional having to interpret what the pain meant and a fear of pain being over-reported [2].

##### Beliefs About Pain Controllability and Pain Management

Children/young people with JIA held differing beliefs about the timeline of their pain both across the day and over time, with some believing their pain would be highest in the morning and reduce throughout the day following walking [6]. Others, including those with a mixed-diagnosis, believed their pain was constant and would get worse over time [18]. Some children/young people with JIA and their parents held the belief that they were able to control their pain, such as through low-intensity physical activity, taking short breaks or pushing through the pain [6]. Healthcare professionals, in particular rheumatologists, believed children/young peoples’ pain could be controlled using pharmacological methods [2]. Children/young people held varying beliefs on their ability to carry on with daily activities despite pain, with some believing they were able to carry on with activities while others were less likely to believe they were capable [18]. Seventy-three percent of children/young people with JRA in one study believed their pain was uncontrollable [1]. Children/young people with JIA and either low disease activity or moderate-high disease activity both shared similar levels of beliefs of pain controllability or belief of the likelihood of there being a medical cure for their pain [7]. Children/young people who reported pain (during a two-week pain diary) compared to those who did not report pain did not differ in the level of their belief of their ability to control pain [3]. Interestingly, children/young peoples’ belief in their ability to control pain can increase over time (two years), including developing a stronger belief in a medical cure [8]. Some children/young people were uncertain about the most appropriate physical exercises to help their pain [18].

#### Noninflammatory Musculoskeletal Conditions

Results below describe children/young peoples’ beliefs about the pain in noninflammatory musculoskeletal conditions (*n* = 8).

##### Beliefs About and Understanding of Pain Cause

Children/young people with complex persistent pain believed they had additional symptoms that were associated with their pain, including limbs changing appearance and sensitivity to heat and/or cold [12]. Boys believed they experienced hyperventilation, fainting, and cramps whereas girls believed they had breathing problems [12]. Children/young people defined a range of potential causes of their pain including minor injury, minor surgery, no specific event or they were uncertain [12]. Parents from a mixed-diagnosis sample reported being uncertain about the cause of pain [17]. Children/young people with a range of noninflammatory diagnoses (e.g., diffuse or localized musculoskeletal pain, CRPS) and their parents were both less likely to believe that tissue damage was always associated with pain however parents were more likely to report a stronger belief that emotions were a cause [15, 16]. Children/young people, including those with juvenile fibromyalgia, CRPS, and other noninflammatory diagnoses [11, 12, 13, 18], believed that other people did not understand or believe their pain, such as friends [11] and healthcare professionals [11, 12, 13, 18]. Some children/young people believed healthcare professionals did not listen to them or take their symptoms seriously [11]. Likewise, children/young people with juvenile fibromyalgia did not always believe in healthcare professionals’ understanding of their pain [11]. For example, children/young people felt that healthcare professionals’ advice and information about their pain could be too narrow by contextualizing pain only in relation to their particular professional specialty [11].

##### Beliefs About the Impact of Pain

Children/young people described a number of consequences they associated with their pain. These included pain negatively impacting their ability to take part in activities [11, 12, 18]; relationships and dynamics with family and friends (resulting in being treated differently, feeling isolated, stigmatized, or forgotten) [11–13]; sleep [12, 18]; quality of life [13]; school attendance [12]; mental health [11]; and having reduced physical functioning [12]. Some children/young people with complex persistent pain felt that their parents suffered more than themselves [12]. Children/young people believed their pain made them feel a range of emotions, including anxiety, fear [11, 12], anger, sadness, worry, frustration [11], despair and depression [12]. Although children/young people with complex persistent pain also reported that these emotions were more prominent in the past, they now held a more accepting view of their pain [12]. Similar emotions were noted in relation to a mixed-diagnosis sample, including children/young people feeling sad and depressed; parents feeling that their child believed their pain was confusing and made them distraught [17]; and that parents believed their child’s pain made themselves (i.e., the parent) feel stressed and depressed [17]. Despite this, children/young people with noninflammatory musculoskeletal conditions still had a positive outlook [12]. Children/young people with a range of noninflammatory musculoskeletal diagnoses (e.g., diffuse, localized, or intermittent musculoskeletal pain or CRPS) [14, 16] or juvenile fibromyalgia [10] had moderate [14] to high pain catastrophizing [10, 16] and parents had moderate pain catastrophizing [16]. Children/young people had significantly higher pain catastrophizing scores than their parents [16]. Children/young people with juvenile fibromyalgia [10], nonspecific chronic low back pain [9], and those from a mixed-diagnosis sample [18] had low [10] to moderate [9] kinesiophobia and described a fear of reinjury [18].

##### Beliefs About Pain Controllability and Pain Management

Children/young people with complex persistent pain believed their pain increased and became worse over time [12]. In relation to mixed-diagnoses, children/young people [18] and parents [17] believed their pain would never go away. Some children/young people with noninflammatory diagnoses believed there was nothing anyone could do to help their pain, including their parents [11]. Similarly, parents from a mixed-diagnosis sample believed that there was nothing that could help their child’s pain [17]. Some children/young people with a range of noninflammatory musculoskeletal diagnoses (e.g., diffuse or localized musculoskeletal pain or CRPS) believed they were able to carry on with daily activities despite pain, while others did not think they were able to [18]. In relation to appropriate pain management, children/young people reported conflicting explanations and advice from healthcare professionals for complex persistent pain [12]. In relation to mixed-diagnoses, parents [17] and some children/young people [18] reported uncertainty on which pain treatment was best.

## Discussion

This is the first scoping review to address how pain beliefs associated with pediatric chronic musculoskeletal conditions have been explored in research to date or what beliefs appear to be closely related to the underlying diagnosis (inflammatory or noninflammatory). The review highlights that heterogeneous methods have been used in the literature to assess pain beliefs. Cross-sectional, quantitative approaches (i.e., using questionnaires) have predominantly been undertaken to date, with less than half of the included studies using theoretical frameworks to examine beliefs. The review identifies interesting similarities and differences in beliefs between musculoskeletal conditions, such as shared views that children/young peoples’ pain is not believed or understood by others. Differences in beliefs between conditions include the perceived cause and controllability of pain. There was more uncertainty surrounding the specific cause of pain in noninflammatory musculoskeletal conditions while disease activity was more likely to be proposed to be a cause of pain in inflammatory musculoskeletal conditions. Pain was deemed only as uncontrollable for noninflammatory diagnoses whereas pain in inflammatory diagnoses was perceived to be controllable to some extent.

To improve understanding of pain beliefs in children/young people with chronic musculoskeletal conditions, research would benefit from assessing beliefs longitudinally in order to understand adaptive changes in experiences, as well as the influence of cognitive and physical development. Beliefs are dynamic representations of health threats that change across time in response to new experiences and/or new information (Leventhal et al., 2012). In only one study to date that conducted a longitudinal study of pain beliefs in children/young people with inflammatory musculoskeletal diagnoses (Thastum & Herlin, 2011), it was found that beliefs about controllability (particularly the likelihood of a medical cure for pain) became stronger over a 2-year period, providing evidence to support that beliefs change over time. Changes in pain beliefs may occur in other conditions such as noninflammatory conditions and other types of pain beliefs may change over time, both of which require further investigation.

Social interactions with healthcare professionals and parents are integral to the formation of children/young peoples’ pain beliefs (Leventhal et al., 1980). Despite this, our scoping review found that healthcare professionals’ beliefs have been largely overlooked, with only one study identified which investigated this key stakeholder perspective. In contrast, the adult literature includes two systematic reviews of healthcare professionals’ beliefs about adults with back pain and found that they were associated with patients’ own beliefs of their pain and healthcare professionals’ clinical management decisions (Darlow et al., 2012; Gardner et al., 2017). For example, healthcare professionals with a stronger biomedical standpoint were more likely to advise patients to limit or delay physical activities which may lead to patients endorsing cautionary and unhelpful beliefs and behaviors toward physical activity (Darlow et al., 2012; Gardner et al., 2017). Future work should explore and compare healthcare professionals’ beliefs about pain associated with pediatric musculoskeletal conditions and whether these influence their clinical advice and management of pain or children/young peoples’ beliefs and behaviors toward their pain.

The associations between pain beliefs and children/young peoples’ health outcomes may benefit from the increased use of theoretical models in research. These provide a useful framework for understanding specific mechanisms by which unhelpful beliefs influence coping behaviors and outcomes (health and pain related). A theory-based, more mechanistic approach would help improve understanding about, and characterization of, pain beliefs which should be targeted and modified, therefore leading to improvements in children/young peoples’ outcomes. As well as the CS-SRM (Leventhal et al., 1980, 2012), there are other prominent cognitive models that provide detailed explanations of pain belief formation and maintenance processes, such as the Fear-Avoidance Model (FAM) (Asmundson et al., 2012; Vlaeyen & Linton, 2000). The FAM proposes that if an individual believes that pain is threatening then this can lead to pain-related fear, avoidance behaviors, and reduced physical and emotional functioning (Asmundson et al., 2012). Notably, single models alone (i.e., the CS-SRM or the FAM in isolation) likely do not holistically capture all nuances and complexities of pain beliefs (Bishop et al., 2015). Less than half of the papers explicitly reported using a theoretical framework and neither the FAM nor CS-SRM were explicitly reported to be implemented. The theories that were used tended to be broad approaches, such as the biopsychosocial approach (Engel, 1980; Engel et al., 2012; Gatchel et al., 2007), which do not provide nuanced descriptions of mechanisms between specific beliefs and outcomes. Researchers should consider the most appropriate theoretical model(s) to address their research question and whether the application of a pre-determined model would instead hinder the exploration of beliefs in this population. For example, the review highlights that further research is necessary to understand what pain beliefs underlie pediatric chronic musculoskeletal conditions. By doing so, this will determine whether, or how, pain beliefs map onto current theories and models.

The review identified a shared belief between musculoskeletal conditions that the legitimacy of pain was questioned, not understood or not believed by others, including healthcare professionals, family, and friends. This has significant clinical, personal, and social implications. Firstly, the questioning of pain legitimacy was particularly noted in inflammatory conditions with well-controlled disease activity but persistent pain. This could pose a risk of healthcare professionals focusing solely on medical management of disease activity and pain, with psychosocial pain management approaches not being provided as an early preventative method, but rather as a reactive approach when medical methods have not worked. In this context, children/young people could interpret this as healthcare professionals believing their pain is solely psychological. Secondly, disbelief of pain and pain dismissal can lead to reduced social functioning and stigmatization (Wakefield et al., 2018), negatively impacted self-esteem and increased hostility (Defenderfer et al., 2018) and feelings of shame and self-blame (Wakefield et al., 2022). It is key that healthcare professionals appropriately assess and manage the range of biological, psychological, and social factors that underpin each individual’s unique pain experience, irrespective of the perceived cause of pain (Clinch & Eccleston, 2009; Stinson et al., 2016). This will allow for earlier identification, prevention, and timely management of children/young people who may be at risk of developing disabling chronic pain conditions or for managing those with a chronic condition (Stinson et al., 2016). Finally, the impact of pain being dismissed or questioned coincides with significant developmental changes during adolescence, including children/young people developing a sense of self, an increased awareness of others’ perceptions, and changes in relationships with parents and friends (Pfeifer & Berkman, 2018). Therefore, the impact of pain dismissal should be considered alongside identity development, particularly as this trajectory is more complex for children/young people with chronic pain conditions. Identity development can be influenced by pain experiences (Eccleston et al., 2008) and from experiencing unique situations associated with their diagnosis (e.g., numerous interactions with healthcare professionals) (Jordan et al., 2018). This can lead to an interrupted or delayed growth of self/identity (Eccleston et al., 2008; Jordan et al., 2018) or, in some instances, earlier emotional maturation (Jordan et al., 2018).

Another belief that is important to understand and assess is pain controllability. This review identified differing levels of controllability between musculoskeletal conditions. Children/young people, parents, and healthcare professionals tended to report some control over pain in relation to inflammatory diagnoses, although one study did find that some children/young people believed their pain was uncontrollable. In contrast, children/young people and parents only reported uncontrollable pain for noninflammatory conditions. Healthcare professionals should assess children/young peoples’ beliefs of pain controllability to understand what degree of control they believe they have, including how to improve their feeling of having more control. The importance of acknowledging this belief is evidenced in other pediatric long-term health conditions (e.g., hypertension, cystic fibrosis, and diabetes), as a higher belief in treatment control is associated with better self-management, such as medication use, diet, and exercise (Law et al., 2014). Similarly, beliefs of low control of pain may benefit from psychological intervention, such as CBT, to allow children/young people to feel they have more personal control over their pain. CBT is a commonly used intervention for pediatric chronic pain which challenges beliefs that pain is uncontrollable and equips the child/young person with coping skills, such as relaxation or pacing (Caes et al., 2018). CBT interventions have been beneficial for improving pain beliefs in children/young people with long-term conditions, including functional abdominal pain, inflammatory bowel disease, and JIA (Levy et al., 2013, 2016; Lomholt et al., 2015).

Notably, this scoping review is the first to our knowledge to consider the categorization of pain beliefs into inflammatory and noninflammatory conditions. Healthcare professionals find this distinction as the most relevant phenotype in clinical practice (Lee et al., 2020), yet no review has explicitly identified, mapped, and compared pain beliefs between these conditions. This review is also strengthened by its application of a theoretical framework. This allowed for a more focused search strategy that highlighted gaps in knowledge and helped to identify future research recommendations, such as how key concepts (i.e., beliefs) in this population map on to current models (Godfrey et al., 2010). The ability to draw conclusions of whether differences in pain beliefs between the conditions were reflections of actual differences or differences in the methods used by the studies was limited. Firstly, it was not possible to provide a descriptive comparison of all questionnaire scores between musculoskeletal conditions due to studies using a heterogeneous range of questionnaires (e.g., the SOPA-C was only used for inflammatory diagnoses) and a lack of consistency between how studies implemented questionnaires within and between condition types (e.g., studies using the same questionnaire but a different number of points on the questionnaire scale). Secondly, it is difficult to identify whether differences in organizational (e.g., healthcare practices), cultural/societal, or ethnic/racial factors influenced these beliefs. For example, there were a range of settings that participants were recruited from (e.g., pediatric rheumatology vs. pain clinics), some noninflammatory conditions but no inflammatory conditions were assessed in the United States, and only six manuscripts provided ethnic/racial information with most participants being White. Further work is necessary to determine the implications of these factors on pain beliefs associated with pediatric chronic musculoskeletal conditions.

To conclude, this scoping review found that highly heterogeneous methods are used to assess pain beliefs underlying pediatric chronic musculoskeletal conditions. The results suggest overlapping beliefs between inflammatory and noninflammatory chronic musculoskeletal conditions, including disbelief of pain by others and impacted functioning. Differences in beliefs between musculoskeletal conditions include the perceived cause of pain and controllability of pain, such as believing disease activity causes pain, and that there is some level of control of pain for inflammatory diagnoses. In contrast, noninflammatory diagnoses are believed to be associated with only uncontrollable pain and the cause of pain is less certain (e.g., minor injury, no known cause). The implications of this review include the need to explore pain beliefs further in this population, including assessing beliefs across time or exploring the role of healthcare professionals’ beliefs, to allow for beliefs that impact children/young peoples’ outcomes (health- or pain-related) to be identified, targeted and modified.

## Supplementary Material

jsad046_Supplementary_Data

## Data Availability

Data are available on request.
